# Analysis of sentinel lymph node biopsy results in colon cancer in regard of the anthropometric features of the population and body composition assessment formulas

**DOI:** 10.1007/s00423-012-0938-4

**Published:** 2012-03-14

**Authors:** Piotr Nowaczyk, Dawid Murawa, Karol Połom, Magdalena Waszyk-Nowaczyk, Arkadiusz Spychała, Michał Michalak, Paweł Murawa

**Affiliations:** 1 1st Clinic of Surgical Oncology and General Surgery, Wielkopolska Cancer Centre, ul. Garbary 15, 61-866 Poznań, Poland; 2 Pharmacy Practice Division, Chair and Department of Pharmaceutical Technology, Poznan University of Medical Sciences, ul. Bukowska 70, 60-812 Poznań, Poland; 3 Department of Computer Science and Statistics, Poznan University of Medical Sciences, ul. Dąbrowskiego 79, 60-529 Poznań, Poland; 4 Department of Oncological Pathomorphology, Wielkopolska Cancer Centre, ul. Garbary 15, 61-866 Poznań, Poland

**Keywords:** Sentinel node biopsy, Colon cancer, Anthropometric features, BMI, Body composition

## Abstract

**Purpose:**

The aim of the study was to assess sentinel lymph node biopsy (SLNB) results in colon cancer (CC) regarding basic anthropometric features of the studied population and their derivatives calculated using mathematical formulas.

**Methods:**

One hundred three SLNBs in CC have been analysed. Various indicators were calculated for every patient using mathematical formulas: BMI, Roher’s index, lean body weight, body fat percentage and body weight/ideal body weight for a given height ratios using the following formulas: Broca’s, Broca’s ideal weight, Broca–Brugsch, Lorenz’s, Potton’s, Devine’s, Robinson’s, Miller’s and Hamwi. The results were compared with accuracy, sensitivity and false negative results percentage by means of ROC curves and the test for structure indicators (for determined cut-off points).

**Results:**

No statistically significant relationship between the results and patients' sex or age were found. ROC curve analysis did not reveal statistically significant relationships between the obtained results and indicators calculated on the basis of growth and weigh (all *p* > 0.05). The analyses of sensitivity and accuracy with determined cut-off point, in spite of differences amounting to 19 % (analysis of lean body weight/weight ratio), showed no statistical significance for any of the relationships (all *p* > 0.05).

**Conclusions:**

No indicator with high diagnostic and prognostic value has been found. The problem of qualifying patients for SLNB in CC in regard of the anthropometric features of the population and body composition assessment formulas remains open and requires further analysis on larger populations.

## Introduction

The most important prognostic factor in the colorectal cancer (CRC) is the local lymphoid system’s status. Lymph node (LN) metastasis lowers 5-year survival rate from 70–90 % to 40–60 % [1–6]. In case of colon cancer (CC) regional lymphoid system involvement decides patient’s eligibility for adjuvant therapy, chemotherapy significantly improves survival rate [2, 6–11]. Up to 30 % of the after surgery pN0 patients will die within 5 years due to local recurrence or remote metastasis [3, 6–13]. As a result, it seems appropriate to establish a “high risk” stage II group that potentially may benefit from adjuvant therapy [1, 3, 7, 8, 14, 15]. Presently, the improvement of diagnosing the LNs seems essential**.** The sentinel lymph node biopsy (SLNB) in CC enables examination of the first nodal station of the lymphatic drainage from the tumour. Precise examination of these LNs using serial tissue slicing, immunohistochemical staining (IHC) and reverse transcription polymerase chain reaction may potentially upstage lymphoid system status compared to the results achieved using haematoxylin and eosin staining (H&E) [3–13, 16, 17]. It needs to be stressed that an en block resection of the tumour with a margin of healthy tissue and mesocolon remains the standard treatment. Recent suggestions to implement SLNB as a mesocolon-sparing treatment during laparoscopic procedures constitute a separate issue [5, 11, 17–19].

Presently, with the ongoing dispute whether to perform SLNB in CC, there’s a need to determine the group of patients who will benefit from it maximally. Qualifying patients means, on one hand, fulfilling certain eligibility criteria and, on the other hand, the issue of stating measurable indicators whether the procedure is legitimate for a given group of patients. Available literature doesn't provide a consensus on qualifying patients regarding basic anthropometric features, such as height, weight and possible derivatives such as Body Mass Index (BMI). Data from papers on SLNB in diseases where it’s a standard procedure (breast cancer, melanoma) suggest there’s a relation between BMI and efficacy and sensitivity of the procedure [20–25]. Analogically, it’s claimed that intra-abdominal obesity may cause difficulties in identifying dyed LNs in the mesocolon. Consequently, it may induce mistakes during the procedure, lowering detection rates or increasing the number of false negative results. This problem affects the most important trend, i.e. SLNB as an extra diagnostic tool for patients with negative H&E staining results of LNs [19, 26, 27]. It’s also influencing the perspectives of using lymph node mapping (LNM) as a mesocolon-sparing treatment.

The aim of this work is to analyse the results of SLNB in CC in regard of basic anthropometric features of the studied population and their derivatives calculated with mathematical formulas. These features may potentially allow assessment of the intra-abdominal obesity and the risk of excessive mesocolon adipose tissue.

## Materials and methods

One hundred three SLNBs in CC have been performed in the 1st Clinic of Surgical Oncology and General Surgery, Wielkopolska Cancer Centre during the period of time from May, 2005 till November, 2010. The studied group consisted of 48 men and 55 women, median age 65 (IQR 56–70)**.** Division of patients according to cancer stage without taking into consideration the IHC examination of sentinel lymph nodes (SLNs) (according to AJCC Cancer Staging Manual, Seventh Edition 2010) is presented in Table 1.

**Table 1:** Division of material with respect to cancer staging according to the TNM, Classification of Malignant Tumours by Dukes and Dukes, modified by Astler and Coller (in accordance with AJCC Cancer Staging Manual, Seventh Edition 2010)

Stage	T	N	M	Dukes	MAC	Patients (%)
0	Tis	N0	M0	–	–	2 (1.9%)
I	T1	N0	M0	A	A	6 (5.8%)
	T2	N0	M0	A	B1	15 (14.6%)
IIA	T3	N0	M0	B	B2	50 (48.5%)
IIIA	T2	N1a	M0	C	C1	1 (1.0%)
	T2	N1b	M0	C	C1	1 (1.0%)
IIIB	T2	N2a	M0	C	C1	2 (1.9%)
	T2	N2b	M0	C	C1	1 (1.0%)
	T3	N1a	M0	C	C2	9 ( 8.7%)
	T3	N1b	M0	C	C2	7 (6.8%)
	T3	N2a	M0	C	C2	5 (4.9%)
IIIC	T3	N2b	M0	C	C2	3 (2.9%)
	T4a	N2a	M0	C	C2	1 (1.0%)

*T* tumour, *N* nodes, *M* metastasis, *Dukes* Dukes staging system, *MAC* Dukes, modified by Astler and Coller, staging system

The criteria of eligibility for the study were: histopathologically confirmed and resectable colon cancer, patient’s age over 18 years old, good overall physical status (ASA I-III), no prior colon or mesocolon surgeries (including appendectomy in case of tumours of the right side of the colon). Patients with intraoperatively assessed suspicious LNs, locally advanced tumours, distant metastases, synchronised neoplasms and operated due to emergency indications (intestinal obstruction) were excluded from the study. All the procedures were open surgeries. Each SNLB was performed by a surgical team including a surgeon who has completed at least ten procedures of this type.

After opening the abdomen in the large intestine, fragment that was to be removed was delicately mobilised in each case. Subserosally, 2–4 ml of Patent Blue dye was administered by four injections. Then pigmentation of lymph vessels and nodes was observed, and sentinel nodes were defined as the first nodes pigmented after 5–10 min from dye application. SLNs were marked with a suture to be subsequently sent for analysis (during or after the procedure) while a standard en block resection of the tumour with a margin of healthy tissue and mesocolon lymphoid system was completed.

Specimen collected during the operation was fixated in a standard way, embedded in paraffin and stained with H&E. If no macrometastases were present, the multisectioning was used for processing the SLNs. The material was cut into sections of the thickness of 250 μm. On each of the obtained levels, at least four sections of 5-μm thickness were stained immunohistochemically (CKMNF116 (Code: M 0821), CKAE1/AE3 (Code: M 3515) monoclonal antibodies, DAKO company, CAM 5,2 (No. 345779) monoclonal antibodies, Becton Dickinson).

SLN was qualified as positive if there was a positive result in H&E staining and if there were micrometastases (foci of cancer cells 0.2–2 mm in diameter) in IHC. LNs containing isolated cancer cells (foci of cells <0.2 mm in diameter) or single cells positive in cytokeratin stain were classified as negative.

To assess efficacy and quality of the procedure, levels of detection, sensitivity and accuracy were calculated. Upstaging, false positive results and the negative predictive value were also calculated. Subsequently, the results were compared to age and sex of the patients. BMI was calculated for every patient using a formula: ${\text{BMI}} = \frac{\text{body weight in kg}}{{{{\left( {\text{height in m}} \right)}^{{2}}}}}$; Roher’s index using a formula: ${\text{Roher}}'{\text{s index}} = \frac{\text{body weight in kg}}{{{{\left( {\text{height in m}} \right)}^{{3}}}}}$; lean body weight using formulas: ${\text{Lean}}\;{\text{Body}}\;{\text{Weight}}\;{\text{for}}\;{\text{men}} = \left( {1.2 \times {\text{body}}\;{\text{weight}}\;{\text{in}}\;{\text{kg}}} \right) - 128\left( {\frac{{{{\left( {{\text{body}}\;{\text{weight}}\;{\text{in}}\;{\text{kg}}} \right)}^2}}}{{{{\left( {100 \times {\text{height}}\;{\text{in}}\;{\text{m}}} \right)}^2}}}} \right),$ ${\text{Lean}}\;{\text{Body}}\;{\text{Weight}}\;{\text{for}}\;{\text{women}} = \left( {1.07 \times {\text{body}}\;{\text{weight}}\;{\text{in}}\;{\text{kg}}} \right) - 148\left( {\frac{{{{\left( {{\text{body}}\;{\text{weight}}\;{\text{in}}\;{\text{kg}}} \right)}^2}}}{{{{\left( {100 \times {\text{height}}\;{\text{in}}\;{\text{m}}} \right)}^2}}}} \right);$ Body Fat Percentage using a formula: Body Fat % = 1.2 × BMI + 0.23 × age in years − 10.8 × (sex; M = 1,F = 0) − 5.4. Body weight for a given height was calculated using Broca’s index: proper weight = height in cm − 100; Broca’s formula for ideal weight: ideal weight F = 0.85 × (height in cm − 100), ideal weight M = 0.9 × (height in cm − 100); Broca–Brugsch formula: ideal weight = height in cm − 100 (for height 155–164 cm) or ideal weight = height in cm − 105 (for height 165–175 cm) or ideal weight = height in cm − 110 (for height 176–190 cm); Lorentz formula for height ≥150 cm: proper weight = height in cm − 100 − 0.25 × (height in cm − 150); Potton’s formula: ${\text{proper weight M}} = {\text{height in cm}} - {1}00 - \left( {\frac{{{\text{height in cm - 100}}}}{{20}}} \right),$ ${\text{proper weight F}} = {\text{height in cm}} - {1}00 - \left( {\frac{{{\text{height in cm - 100}}}}{{10}}} \right)$. We also calculated body weight using formulas known in drugs dosage calculations: Devine’s formula: ideal weight M = 50 + 2.3 kg for every inch over 5 ft, ideal weight F = 45.5 + 2.3 kg for every inch over 5 ft; Robinson’s formula: ideal weight M = 52 + 1.9 kg for every inch over 5 ft, ideal weight for women = 49 + 1.7 kg for every inch over 5 ft; Miller’s formula: ideal weight M = 56.2 + 1.41 kg for every inch over 5 ft, ideal weight F = 53.1 + 1.36 kg for every inch over 5 ft; Hamwi formula: ideal weight M = 48 + 2.7 kg for every inch over 5 ft, ideal weight F = 45.5 + 2.2 kg for every inch over 5 ft. The result from every weight formula was matched to patient’s current weight: current weight/ideal weight according to a given formula.

Results for each sex were analysed using chi-square test, for every variable depending on sex, age, mass and height, ROC graphs were plotted to find cut-off points for sensitivity, accuracy, and false negative results percentage in SLNB in CC. Additionally, cut-off points were determined for particular calculated parameters: for BMI, 23 and 25; for lean body weight, 0.72; for body fat percentage, 28 %; for all the ratios current weight/ideal weight for a given height, 1. The obtained results were analysed in terms of method sensitivity and accuracy, each single time comparing two groups on the both sides of a cut-off point with the use of the test for structure indicators.

The study has been approved by the Bioethics Commission of Poznan University of Medical Sciences.

## Results

In an average specimen, 14.3 LNs were analysed (median 13, IQR 9.75–18). In 66 % (68) of patients 12 or more LNs were analysed. No complications during SLNB procedure were observed and at least one SLN (median 3, IQR 2–4) was identified in 102 out of 103 patients (99 %). After H&E and IHC staining, the SLN was confirmed as the only place of metastasis in 13/102 patients (12.7 %). Accuracy of SLN in predicting the status of the local lymphatic system was 94.1 % (96/102). Sensitivity of the method in predicting the status of the local lymphatic system was 84.2 % (32/38). The index of false negative results was 15.8 % (6/38). In three out of these six patients, aberrant lymphatic drainage (ALD) was observed after administering dye; mesenteric root LN were marked. ALD percentage was 2.9 % (3/102). Negative predictive value was 91.4 % (64/70). In seven patients with negative SLN after H&E staining and its positive result in IHC stain, no tumour deposits were found in the remaining specimen, so the upstaging due to IHC stain was 9.9 % (7/71).

Median height in the population was 1.68 m (IQR 1.60–1,72 m). Median body weight was 72 kg (IQR 61.5–81.25). Median BMI was 25.64 kg/m^2^ (IQR 22.63–28.33 kg/m^2^). There was no statistically significant difference in the results regarding sex and age of the patients. In the plotted ROC graphs, there was no significant relation for all of the indicators calculated based on body weight and height. In the studied material, height/weight ratio (area under curve—AUC = 0.50, *p* = 0.4771), BMI (AUC = 0.53, *p* = 0.3223), Roher’s index (AUC = 0.54, *p* = 0.2340), lean body weight/weight ratio (AUC = 0.42, *p* = 0.9258), body fat percentage (AUC = 0.57, *p* = 0.1171) and the ideal weight calculated using Broca’s (AUC = 0.54, *p* = 0.2790), Broca’s ideal weight (AUC = 0.54, *p* = 0.2441), Broca–Brugsch (AUC = 0.51, *p* = 0.4123), Lorenz’s (AUC = 0.48, *p* = 0.6153), Potton’s (AUC = 0.54, *p* = 0.2494), Devine’s (AUC = 0.55, *p* = 0.2216), Robinson’s (AUC = 0.55, *p* = 0.2088), Miller’s (AUC = 0.53, *p* = 0.3112) and Hamwi (AUC = 0,56, *p* = 0.1651) formulas were all insignificant.

The results of sensitivity and accuracy obtained in particular groups for the determined cut-off points are presented in Tables 2 and 3. In spite of differences in the sensitivity and accuracy of the method, reaching 19 % and obtained in the calculations for the particular groups, statistical significance was not found for any of the parameters.

**Table 2:** Sensitivity results obtained in particular groups for the determined cut-off points

BMI	<25	≥25	*p* value
	75%	91%	0.1811
	<23	≥23	*p* value
	80%	86%	0.6534
Lean body weight/body weight	≥0.72	<0.72	*p* value
	94%	75%	0.1107
Body Fat Percentage	<28%	≥28%	*p* value
	100%	82%	0.3542
Weight/Broca weight for a given height	<1.00	≥1.00	*p* value
	85%	84%	0.9359
Weight/ideal weight Broca	<1.00	≥1.00	*p* value
	100%	83%	0.5244
Weight/ideal weight Broca–Brugsch	<1.00	≥1.00	*p* value
	80%	86%	0.6534
Weight/ideal weight Loretz	<1.00	≥1.00	*p* value
	78%	86%	0.5864
Weight/ideal weight Potton	<1.00	≥1.00	*p* value
	86%	84%	0.8953
Weight/ideal weight Devine	<1.00	≥1.00	*p* value
	100%	82%	0.3542
Weight/ideal weight Robinson	<1.00	≥1.00	*p* value
	80%	85%	0.7743
Weight/ideal weight Miller	<1.00	≥1.00	*p* value
	88%	83%	0.7314
Weight/ideal weight Hamwi	<1.00	≥1.00	*p* value
	89%	83%	0.6646

**Table 3:** Accuracy results obtained in particular groups for the determined cut-off points

BMI	<25	≥25	*p* value
	91%	96%	0.2987
	<23	≥23	*p* value
	93%	95%	0.6938
Lean body weight/body weight	≥0.72	<0.72	*p* value
	98%	92%	0.1919
Body Fat Percentage	<28%	≥28%	*p* value
	100%	93%	0.2153
Weight/Broca weight for a given height	<1.00	≥1.00	*p* value
	95%	93%	0.6812
Weight/ideal weight Broca	<1.00	≥1.00	*p* value
	100%	94%	0.476
Weight/ideal weight Broca–Brugsch	<1.00	≥1.00	*p* value
	92%	95%	0.571
Weight/ideal weight Loretz	<1.00	≥1.00	*p* value
	91%	95%	0.4738
Weight/ideal weight Potton	<1.00	≥1.00	*p* value
	94%	94 %	1
Weight/ideal weight Devine	<1.00	≥1.00	*p* value
	100%	93%	0.3648
Weight/ideal weight Robinson	<1.00	≥1.00	*p* value
	92%	94%	0.7809
Weight/ideal weight Miller	<1.00	≥1.00	*p* value
	96%	94%	0.7077
Weight/ideal weight Hamwi	<1.00	≥1.00	*p* value
	96%	93%	0.585

## Discussion and conclusions

The influence of anthropometric factors on SLNB is treated marginally in literature. The vast majority of available data concerns breast cancer where mostly isotope and dye double technique is used. Radiocolloid is used very rarely in SLNB in CC (it migrates faster in colon LVs than in breast or melanoma, thus increasing the probability of marking more hot sentinel nodes; also, identification is harder due to closeness of the primary tumour and the node and the radiation overlap) [13, 26, 28–30]. Nevertheless, in view of general study ideas and lack of reports, the problem seems worth mentioning. In a multi-centre British study ALMANAC [20] on SLNB in breast cancer, there was a relation found between BMI and detection rate—the group with BMI ≤ 30 kg/m^2^ (*n* = 425) showed statistically higher detection rate than the group with BMI > 30 kg/m^2^ (*n* = 113) (98.1 % vs. 87.6 %, *p* < 0.001). The relation was not confirmed for the percentage of false negative results. Another study analysing 748 SLNBs in breast cancer [21] has shown that SLN identification failure rate was 15.9 % for patients with BMI > 27 kg/m^2^, 8.5 % for patients with BMI of 22.5–27 kg/m^2^ and 7.7 % for patients with BMI < 22.5 kg/m^2^. This study additionally stated that high BMI not only reduces tissue translucency but also that excessive fat may pressurise LV walls interfering with proper drainage. An American study [22] analysing 2,495 SLNBs in breast cancer using isotope-dye technique demonstrated inverse relation between BMI and identification frequency (*r* = −0.98, *p* = 0.002). This relation was statistically significant in groups under and over 50 years old. An average BMI was compared in groups with positive and negative identification showing statistically significant difference (25.9 vs. 29.4, *p* < 0.001) both in groups under and over 50 years old. A multifactorial analysis stated that age and BMI have the most significant (inversely proportional) influence on LNM efficacy. Similar results were obtained also in some other studies on the relationship between the results of sentinel node biopsy in breast cancer and BMI [23–25].

Currently, there are only a few available studies on qualifying patients for SLNB in CC. There weren’t any satisfactory attempts to discuss qualifying patients for the procedure in regard of anthropometric features. Both a meta-analysis by Des Guetz et al. [4], a review article by Bembenek et al. [28] and the most recent meta-analysis by van der Pas et al. [31] do not raise the problem of qualification of patients in terms of anthropometric features. The issue has been addressed in a work by Cahill et al. [19]—the authors found data on BMI only in four papers included in the meta-analysis. A relationship between high BMI and intra-abdominal obesity has been potentially determined as a factor that disrupts mesocolon translucency, hinders identification and increases false negative results rate in SLNB in CRC and, additionally, that causes difficulties for a surgeon. Some of the studies available for in-vivo mapping are mentioned in Table 4. They utilise simplest derivatives of height and weight, both of which are practically always measured on admittance. The question remains whether BMI is a good representation of intra-abdominal obesity which possibly may interfere with identifying marked LNs in the mesocolon. Undoubtedly, excessive adipose tissue in the mesocolon potentially causes mistakes during the procedure, i.e. lowers detection rates or increases number of false negative results. This conclusion is, however, based on the authors' experience, not EBM data.

**Table 4:** Studies of SLNB in CC already published and discussing the problem of anthropometric features of the population

Main author	Year	Published in	Method	Number of patients	BMI (average) (kg/m^2^)	BMI (range) (kg/m^2^)	Detection rate (%)	Sensitivity (%)	False negative results (%)	Conclusion
Viehl	2003	World J Surg	Isosulfan blue	31	25.3	19.5–38.3	87	50	22	No statistically significant relation between detection rate, sensitivity, % of false negative results and BMI
Thomas	2006	Ann Surg	Isosulfan blue	69	circa 26.3	–	93	46	54	No statistically significant relation between detection rate, % of false negative results and BMI (cut-off point BMI = 25 kg/m^2^)
Tiffet	2007	Dis Colon Rectum	Radiocolloid Patent blue	49	25	14–34	92	80	20	No statistically significant relation between detection rate and BMI (cut-off point BMI = 30 kg/m^2^); % of false negative results much higher in patients with BMI ≥ 30 kg/m^2^ (18 % vs. 33 %); due to small number of analysed patients statistical significance was not assessed
Bembenek	2007	Ann Surg	Patent blue	315	–	–	85	54	46	Detection rate statistically significantly lower In patients with BMI > 25 kg/m^2^ (92.5 % vs. 80 %, *p* = 0.002); Sensitivity statistically significantly higher for patients with BMI ≤ 24 kg/m^2^ (80 % vs. 42 %, *p* < 0.0001), as a result lower % of false negative results
Cahill	2009	Ann Surg Oncol	Isosulfan blue Patent blue	225	26.6	23.4–30.6	95	97	3	BMI as a variable in calculating sensitivity rates made no notable difference (both in ROC analysis and around arbitrary cut-off at 24 kg/m^2^)

In the study of Thomas et al. [32], an assumption was made that thickened mesocolon containing excessive adipose tissue may impede with identifying LNs. An obligatory division of patients into groups was made in the analysis, based on calculated BMI: underweight patients, BMI < 18.5 kg/m^2^; normal weight patients, BMI 18.5–24.9 kg/m^2^; overweight patients, BMI 25–29.9 kg/m^2^; obese patients, BMI > 30 kg/m^2^. In the group of 61 (out of 69 included in the study), there was no statistically significant difference found in precision of LNM nor the number of false negative results comparing the group of underweight patients and those with normal weight with the group of overweight and obese patients. It was concluded that BMI alone does not inform about body fat distribution—patients with the same BMI may have different amounts of mesocolon fat.

Another study [33] analysed 31 patients with average BMI of 25.3 kg/m^2^ (19.5–38.3). There were no statistically significant differences in BMI found between groups with positive and negative identification of SLN. In the study of Tiffet et al. [26], an assumption was made that obesity determined by BMI is a potential reason of detection failure. However, there was no statistically significant relation between detection rates and patients' BMI. It was determined that sensitivity was higher (53 % vs. 40 %) and false negative results percentage lower (18 % vs. 33 %) in patients with BMI < 30 kg/m^2^, but no statistical significance was shown.

A study by Cahill et al. [18] that examined the available data bases from a multi-centre German study [27] and material by Saha et al. [8], analysed in detail the results of SLNB for early changes—T1 and T2. The relationship between sensitivity and BMI was analysed both with the use of ROC and the arbitrary cut-off point—24 kg/m^2^. No statistically significant relationship was demonstrated in any of the analyses. However, it is emphasised in the Discussion section that including BMI in the exclusion criteria, hypothetically, is one of the ways of increasing the procedure sensitivity.

Similarly to the four above-mentioned studies, our research in ROC analysis did not show any relation between BMI and SLNB in CC results. Also, in the analyses conducted for arbitrary cut-off points (BMI of 23 and 25), no statistically significant relationships were found; what may be found puzzling is that better results for sensitivity and accuracy were obtained for the patients with higher BMI.

In a multi-centre German study [27], the examined population consisted of 315 patients. It concluded that a statistically higher detection rate was achieved in a group with BMI ≤ 25 kg/m^2^ as compared to the group with BMI > 25 kg/m^2^ (92.5 % vs. 80 %, *p* = 0.003). Additionally, it was proven for the examined population that there is a statistically significant inverse relation between BMI and sensitivity (*p* = 0.009). This relation was found in ROC graph analysis comparing groups with BMI ≤ 24 kg/m^2^ (sensitivity 80 %) and BMI > 24 kg/m^2^ (sensitivity 42 %) (*p* < 0.0001). Furthermore, it was observed that SLNB in patients with BMI ≤24 kg/m^2^ conducted in centres with more experience, the sensitivity was as high as 88 %. The summary underlined LVs visualisation problem in obese patients and the influence of BMI on achieved SLNB results.

In summary, our research has not proved any statistically significant indicator to have high diagnostic and prognostic value. There was no statistical significance shown for BMI—value which is most frequently examined. Also, we failed to demonstrate significance of any other indicator in spite of differences in the sensitivity and accuracy of lymphatic mapping, running into 19 % for some parameters. Thus, the problem of qualifying patients for SLNB in CC remains open and requires further analysis on larger populations.
